# Colocalization of Coregulated Genes: A Steered Molecular Dynamics Study of Human Chromosome 19

**DOI:** 10.1371/journal.pcbi.1003019

**Published:** 2013-03-28

**Authors:** Marco Di Stefano, Angelo Rosa, Vincenzo Belcastro, Diego di Bernardo, Cristian Micheletti

**Affiliations:** 1 International School for Advanced Studies (SISSA), Trieste, Italy; 2 Philip Morris International R&D, Philip Morris Products S.A., Neuchâtel, Switzerland; 3 Telethon Institute of Genetics and Medicine (TIGEM), Napoli, Italy; 4 Department of Informatics and Systems Engineering, University “Federico II”, Napoli, Italy; University of British Columbia, Canada

## Abstract

The connection between chromatin nuclear organization and gene activity is vividly illustrated by the observation that transcriptional coregulation of certain genes appears to be directly influenced by their spatial proximity. This fact poses the more general question of whether it is at all feasible that the numerous genes that are coregulated on a given chromosome, especially those at large genomic distances, might become proximate inside the nucleus. This problem is studied here using steered molecular dynamics simulations in order to enforce the colocalization of thousands of *knowledge-based* gene sequences on a model for the gene-rich human chromosome 19. Remarkably, it is found that most (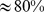) gene pairs can be brought simultaneously into contact. This is made possible by the low degree of intra-chromosome entanglement and the large number of cliques in the gene coregulatory network. A clique is a set of genes coregulated all together as a group. The constrained conformations for the model chromosome 19 are further shown to be organized in spatial macrodomains that are similar to those inferred from recent HiC measurements. The findings indicate that gene coregulation and colocalization are largely compatible and that this relationship can be exploited to draft the overall spatial organization of the chromosome *in vivo*. The more general validity and implications of these findings could be investigated by applying to other eukaryotic chromosomes the general and transferable computational strategy introduced here.

## Author Summary

Recent high-throughput experiments have shown that chromosome regions (*loci*) which accommodate specific sets of coregulated genes can be in close spatial proximity despite their possibly large sequence separation. The findings pose the question of whether gene coregulation and gene colocalization are related in general. Here, we tackle this problem using a knowledge-based coarse-grained model of human chromosome 19. Specifically, we carry out steered molecular dynamics simulations to promote the colocalization of hundreds of gene pairs that are known to be significantly coregulated. We show that most () of such pairs can be simultaneously colocalized. This result is, in turn, shown to depend on at least two distinctive chromosomal features: the remarkably low degree of intra-chain entanglement found in chromosomes inside the nucleus and the large number of cliques present in the gene coregulatory network. The results are therefore largely consistent with the coregulation-colocalization hypothesis. Furthermore, the model chromosome conformations obtained by applying the coregulation constraints are found to display spatial macrodomains that have significant similarities with those inferred from HiC measurements of human chromosome 19. This finding suggests that suitable extensions of the present approach might be used to propose viable ensembles of eukaryotic chromosome conformations *in vivo*.

## Introduction

The advent of innovative fluorescence-based techniques has provided an unprecedented insight into the organization of eukaryotic chromosomes during various phases of the cell cycle [1], [2]. A notable example is given by the demonstration - based on imaging techniques - that when the tightly packed mitotic chromosomes enter interphase they swell and occupy specific nuclear regions, aptly termed “territories” [1]. More recently, the salient local and global spatial properties of chromatin fibers inside these territories have been addressed by the so-called “chromosome conformation capture” techniques [3]–[7], which allow for probing the cis/trans contact propensity of various chromosomal *loci*.

The recent systematic application of these experimental techniques is providing increasing evidence that chromosomes are organized in functionally-heterogeneous macrodomains with different molecular and genetic composition [6],[8],[9].

Several efforts are being spent to clarify the functionally-oriented implications of such chromosomal organization. Towards this goal, some of us have recently carried out a comprehensive bioinformatic survey of data gathered in more than 20,000 gene expression profiles measured for several cell lines in different human tissues [10]. It was thus established that genes can be grouped into large clusters based on significant pairwise correlations (mutual information) of their expression patterns. In addition, the matrix of pairwise gene expression correlations displayed features qualitatively similar to the matrix of pairwise gene contacts inferred from the HiC [6].

Furthermore, for various model organisms, specific sets of genes that are systematically coexpressed were shown to be in spatial contact too [11]–[13]. A chief example is provided by the human 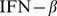 gene, an 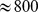 base pairs-long region on human chromosome 9. This gene, during virus infection, induces colocalization and coexpression of 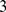 distant 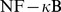 bound genomic *loci* [14].

While not all sets of coexpressed or coregulated genes are expected to be nearby in space [15], several arguments and model calculations have consistently indicated that the simultaneous colocalization of multiple genes can occur with appreciable probability even when the genes are far apart along a chromosome and in the presence of a crowded nuclear environment [16],[17]. Indeed, it has been argued that the cooperative colocalization of various genes can provide a very efficient means for achieving their functional coregulation [18],[19].

These considerations motivated the present numerical study where a knowledge-based coarse-grained model of eukaryotic chromosome 19 is used to ascertain whether the large number of coregulated gene pairs on a given chromosome can be actually colocalized in space. The analysis therefore complements recent efforts through which the organization of model chromatin fibers was investigated by bringing distant regions into contact by using attractive interactions, which either mimicked the effect of transcription factories [17] or 5C-based distance restraints [20].

Our investigation, is carried out for human chromosome 19 (Chr19). This chromosome, which is typically located at the nucleus center [6], was chosen because it has the highest gene density and extensive gene expression data are available for it. By analysing the mutual information content of thousands of such expression profiles we identify hundreds of coregulated gene pairs for Chr19. These coregulated gene pairs are next mapped onto a previously-validated model for interphase chromosomes (where the chromatin filament is coarse-grained at a resolution of 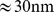) and their pairwise colocalization is enforced using a steered molecular dynamics scheme. The protocol is applied to various initial chromosome configurations where the degree of entanglement is comparable to that expected for chromosomes *in vivo* (based on the crumpled-globule interpretation of HiC data [6],[21]) or much higher (as in equilibrated polymer chains). Further terms of comparisons were obtained by randomizing the positions or pairings of the *loci* to be colocalized.

Notably, for initial chromosome conformations with low entanglement, it is found that most (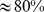) of the coregulated gene pairs can indeed be brought into contact and this promotes the formation of spatial macrodomains similar to those inferred from HiC measurements of human chromosome 19. The percentage of satisfied colocalization constraints, and the macrodomain similarity is dramatically reduced when the initial chromosome arrangements are significantly entangled and when the coregulatory network is changed by suppressing the numerous native coregulatory cliques, that are groups of genes all mutually coregulated.

The observed compliance of the model chromosomes towards the gene colocalization demonstrates that bringing into simultaneous spatial proximity most of the thousands of coregulated gene pairs for Chr19 is physically viable. The findings are therefore consistent with the hypothesis that coregulated genes are likely to be in contact too. This conclusion is further supported by the fact that the spatial macrodomains found in the constrained, steered conformations of Chr19 are well-consistent with those inferred from Hi-C data.

## Results/Discussion

### Colocalization of coregulated genes in human chromosome 19

A number of experimental studies have given the consensual indication that various sets of coregulated genes tend to be nearby in space, even if they are at a large genomic distance (reviewed in Ref. [12]). Because gene colocalization is not necessary in principle to achieve gene coregulation or coexpression (the latter can, for instance, be induced by controlled hormone addition [15]) it is not clear whether there exists a general connection between gene coregulation and gene colocalization and what would be the general biological implications.

In particular, two such important ramifications regard the interplay of chromosome conformational arrangement and gene expression or regulation. The first issue relates to the entanglement of the long and densely packed chromatin filaments: is their arrangement too intricate to allow for the simultaneous colocalization of all (or most) pairs of coregulated genes? Secondly, in case there exists a strong association between gene coexpression and colocalization, is it at all feasible to use gene coexpression data as distance restraints to pin down viable chromosome conformations?

To make progress on these standing issues we developed and used a knowledge based numerical approach to investigate the gene coregulation–colocalization relationship in human Chr19 using a coarse-grained chromosome model.

Chr19 which is 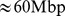 long, was chosen because it has the highest gene density compared to other chromosomes [22]. This property reflects, in turn, in the possibility to use publicly available gene expression data to derive knowledge based colocalization constraints that cover extensively Chr19.

To this purpose we started by considering 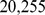 expression measurements for 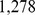 probe sets for Chr19. As customary we shall hereafter refer to the probe sets simply as genes. By analysing this large pool of data using the approach described in the Materials and Methods section, we singled out 1,487 pairs of genes which, according to the high mutual information content of their expression profiles, are deemed to be significantly coregulated [23].

Notably, the selected pairs of genes are typically far apart along the chromosome contour. The median genomic separation of the midpoints of the coregulated genes is as large as 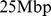.

To clarify whether, and to what extent, the coregulated gene pairs can be simultaneously colocalized we used a coarse-grained model for chromatin filaments that has been previously shown to be capable of accounting for the fractal-like organization observed for eukaryotic chromosomes [6],[21],[24]–[28]. Specifically, we adopted the model of Ref. [21] where chromatin is described as a homogeneous chain of beads with effective diameter equal to 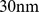 and persistence length equal to 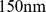. Accordingly, Chr19 is described as a chain of 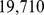 beads, for a total contour length of 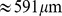.

**Figure 1:**
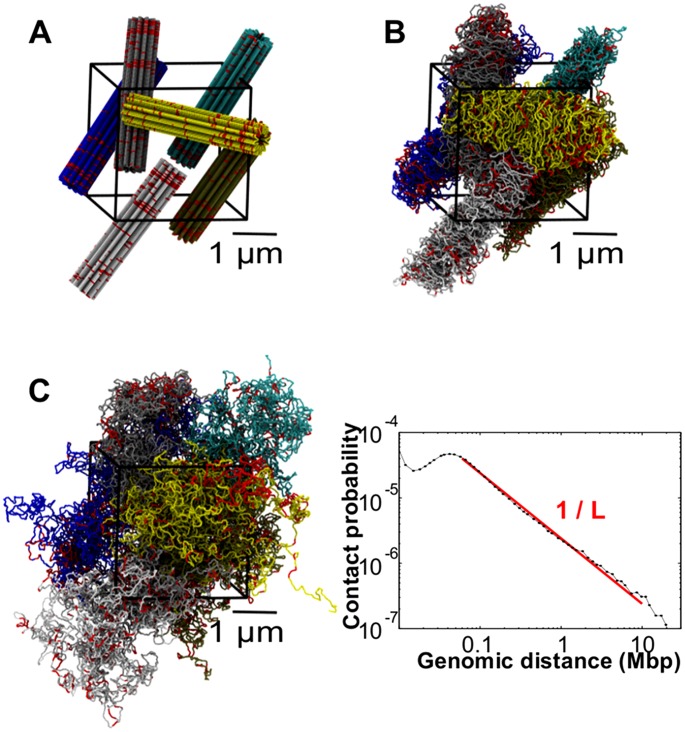
Mitotic and interphase configurations of the model system chromosomes. (A) Initial mitotic-like arrangement, constituted by 6 copies of model human chromosome 19. Following ref. [21], the chromatin fiber is helicoidally arranged into loops of 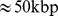 each, and departing radially from a central axis. The six solenoidal arrangements were next placed in a random, but non-overlapping manner inside a cubic simulation box of side equal to 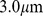 and with periodic boundary conditions. (B) Chromosome spatial arrangement after short relaxation with a standard push-off protocol of 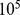 MD time steps (see Materials and Methods). (C) Interphase-like configuration obtained by evolving the initial mitotic configuration for 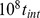 MD time steps (approximately corresponding to 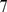 hours in “real-time” [21]). (*Inset*) The corresponding contact probabilities between *loci* of model interphase chromosomes decay as a power law of the genomic distance, 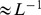, consistent with recent experimental observations [6],[29]. In all panels, chromosome regions involved in the coregulatory network are highlighted in red.

To mimic inter-chromosome interactions in the dense nuclear environment, we considered a system where six copies of Chr19 are placed in a cubic simulation box (with periodic boundary conditions) of side equal to 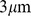. The overall system density is therefore 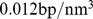, which corresponds to a 10% volume fraction. Such density matches the typical genomic one in human cell nuclei (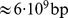 in a nucleus that is 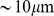 in diameter [21]). To mimic the mitotic state, each model chromosome was initially prepared in an elongated solenoidal-like configuration [21], and the six copies were placed in a random, but non-overlapping arrangement inside the cubic simulation box as shown in Fig. 1A. To remove any excessive intra-chain strain of the orderly designed mitotic arrangement, the model chromosomes of Fig. 1A were briefly evolved with an unbiased MD protocol. The resulting relaxed mitotic configuration is shown in Fig. 1B.

This mitotic arrangement was further evolved for a much longer simulation time, roughly corresponding to 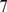 hours in “real-time” [21], to obtain the fully decondensed arrangement shown in Fig. 1C. Such configuration exhibits the same power-law decay of contact probabilities versus genomic separation as observed in HiC experiments [6],[29], see inset of Fig. 1C. The model system therefore aptly reproduces the salient experimentally-observed features of interphase chromosomes.

After setting up the mitotic and interphase systems, we next applied a steered molecular dynamics protocol to each of them (see Materials and Methods) to promote the spatial proximity of regions corresponding to coregulated gene pairs.

**Figure 2:**
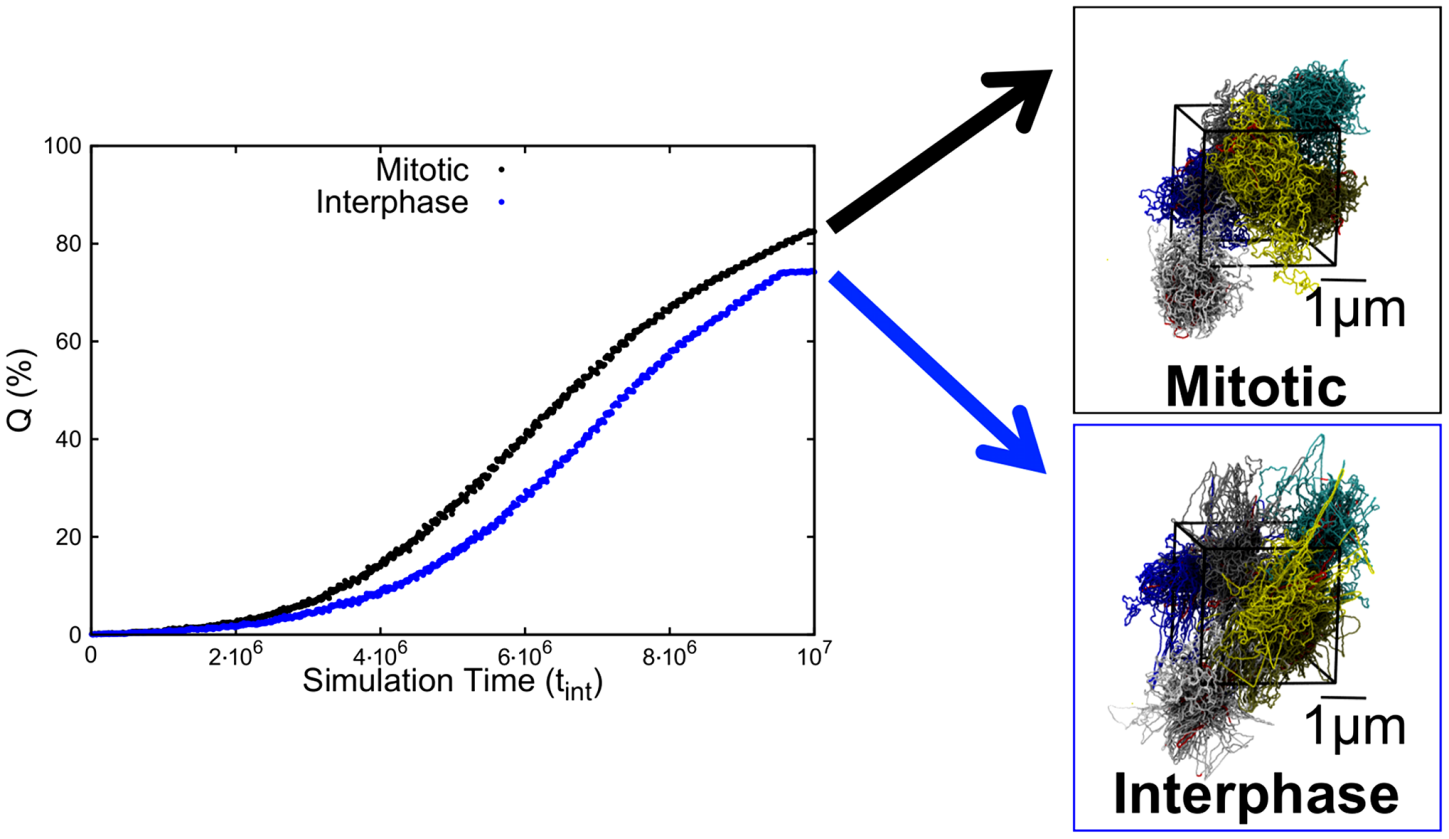
Increase of the percentage, 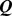, of Chr19 coregulated pairs which colocalize during the MD steering protocol. The two curves reflect different initial conditions corresponding to the mitotic and the interphase conformations of panels (B) and (C) of Fig. 1. The final configurations, corresponding to 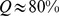 are shown on the right. Chromosome regions involved in the coregulatory network are highlighted in red. These and other graphical representations of model chromosomes were rendered with the VMD graphical package [47].

The compliance of the two systems to the steering protocol is illustrated in Fig. 2 which shows the increase of the percentage of target gene pairs that are successfully colocalized.

It is striking to observe that for both system it is possible to simultaneously colocalize a very high fraction of the target pairs, namely 80% of them (averaged over the six chromosome copies). The conformations reached at the end of the steering protocol are shown in the right panels of Fig. 2.

Considering the relatively-high density of the simulated system of chromosomes and that most of the coregulated pairs lie at large genomic distances, the results point to an unexpectedly high degree of plasticity of the mitotic and interphase conformations, which is presumably ascribable to their fractal-like metric properties which keeps at a minimum the entanglement of the chromatin fiber [6],[21],[24]–[28],[30].

A second noteworthy feature of the results of Fig. 2 emerges considering the diversity of the sources used to derive the knowledge-based coregulation data. In fact, granted the validity of the coregulation–colocalization hypothesis, one might have envisaged *a priori* that the chromosomal configurations corresponding to different tissues or experimental conditions would be so heterogeneous that it would be impossible to satisfy the cumulated set of colocalization constraints. By contrast, the results of Fig. 2 demonstrate *a posteriori*, that the set of pairwise colocalization constraints are largely mutually compatible because most of them can be simultaneously satisfied.

The findings are therefore not only consistent with the coregulation–colocalization hypothesis but, based on such hypothesis, also suggest that the conformations adopted by a chromosome in various conditions can share a common underlying pattern of colocalized genes.

### Spatial macrodomains: comparison with data based on HiC maps

To further characterize the overall organization of the steered conformations shown in Fig. 2 we identified their spatial macrodomains and compared them with those inferred from the analysis of HiC data collected by Dixon *et al.* [9].

**Figure 3:**
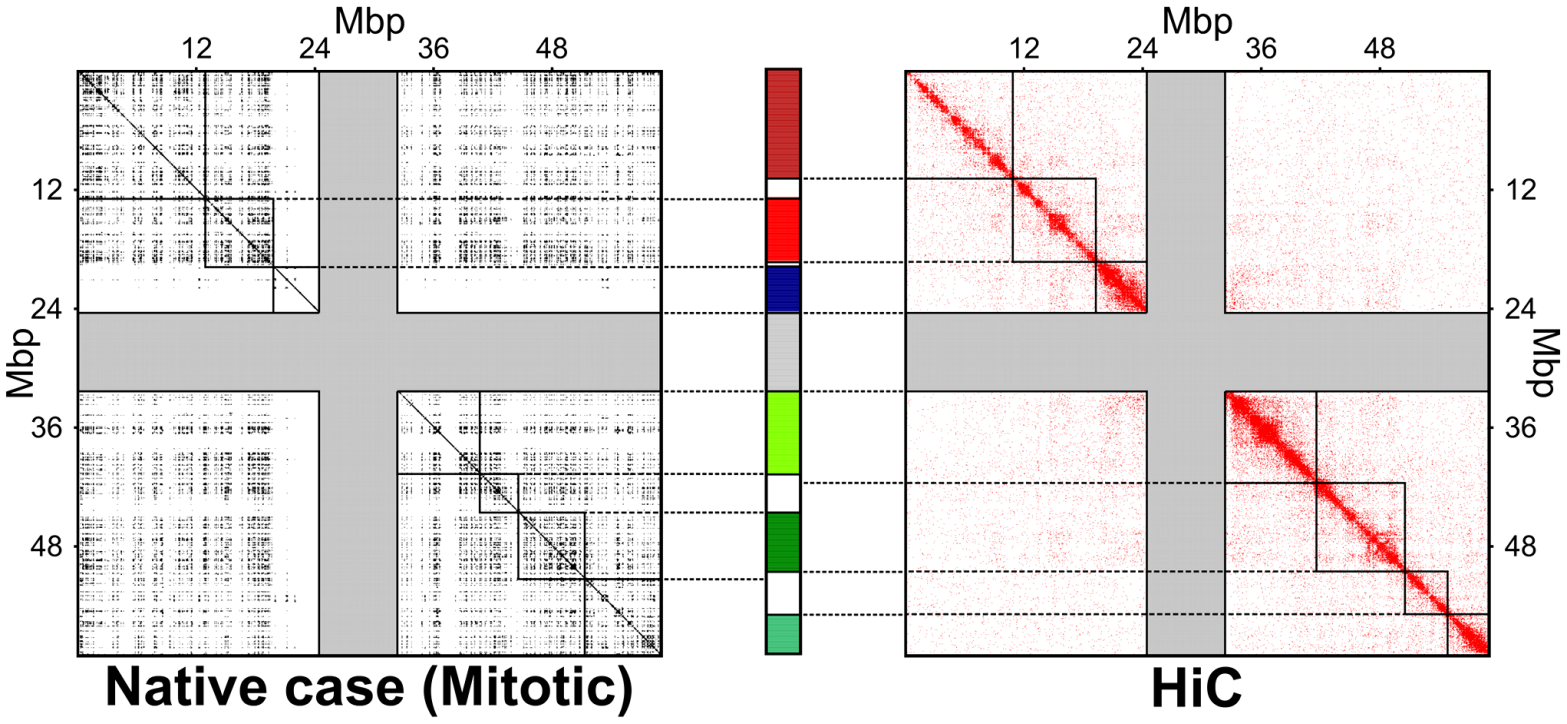
Spatial macrodomains. The contact maps for Chr19 obtained at the end of the steered-MD simulations and inferred from HiC data are shown on the left and right, respectively. The grey bands mark entries involving the centromere region. The boundaries of the 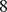 principal spatial domains, identified with a clustering analysis of the contact maps, are overlaid on the matrices. The consistency of the two macrodomain subdivisions is visually conveyed in the chromosome sketch at the center. The overlapping portions of the domain subdivisions are colored (different colors are used for different domains). Non-overlapping regions are shown in white, while the centromere region is shown in grey. The overlapping regions accounts for 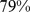 of the chromosome (centromere excluded).

In both cases, the starting point of the analysis was the construction of the chromosome contact map with a 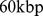 resolution, which is commensurate with both the experimental resolution (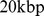) and the bead equivalent contour length (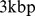). The HiC-data based contact map was derived from the contact enrichment values reported by Dixon *et al.* [9] while the simulation-based one was computed from the bead pairwise distances at the end of the steering protocol (averaged over the six chromosome copies), see Materials and Methods. Both matrices are shown in Fig. 3.

A clustering analysis of the contact maps was next used to subdivide Chr19 into up to ten spatial macrodomains, each spanning an uninterrupted chromosome stretch, and with the proviso that one domain should cover the centromere. For both maps the consensus domain boundaries were well-captured by the subdivision into eight spatial domains, see Fig. S1. The corresponding macrodomain partitions are overlaid on the contact maps of Fig. 3.

The good consistency of the domains found using HiC-based and steered-MD contacts maps is visually conveyed by the matching colored regions in the schematic chromosome partitioning of Fig. 3. It is interesting to notice that the two domain subdivisions consistently indicate larger domains for the upper arm. Quantitatively, the overlap of the two subdivisions is 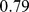, which has a 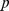-value smaller than 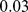. This means that random partitions of the chromosome into eight domains (one always being the centromere) yields overlaps 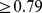 in less than 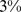 of the cases, see Fig. S2. The quantitative comparison therefore indicates a statistically-significant consistency of the spatial macrodomains arising in the steered chromosome conformations and those inferred from experimental data.

### Chromosome entanglement, regulatory network properties and gene colocalizability

Besides the previous considerations, the results of Fig. 2 prompt the question of whether, and to what extent the feasibility to colocalize a significant fraction of the coregulated gene pairs depends on distinctive chromosomal features, such as the spatial arrangement of the mitotic and decondensed states or the network of coregulated genes.

**Figure 4:**
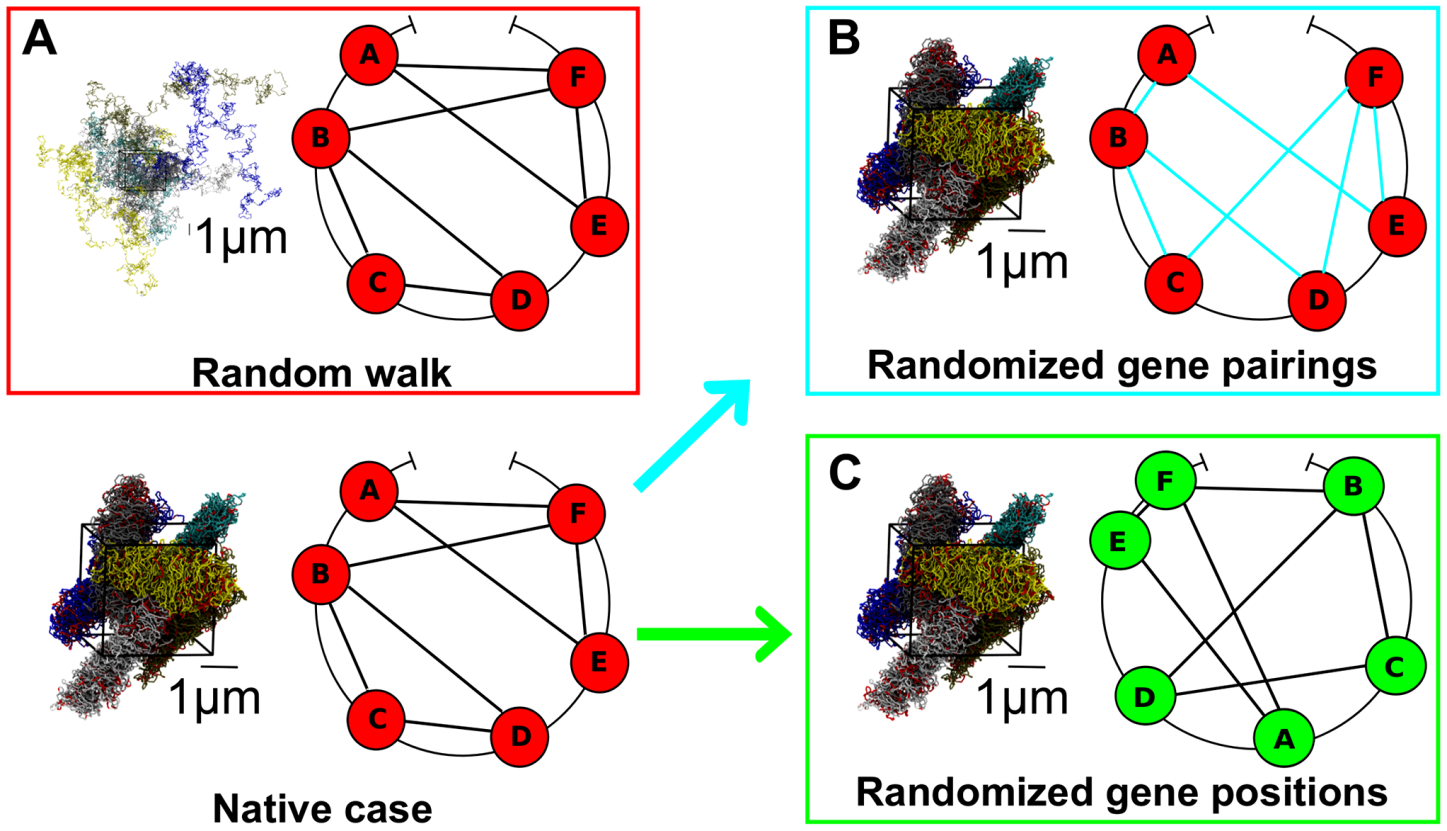
Variant systems subjected to the MD steering protocol. (A) Initial configuration of 6 random-walk like chains the linear size the model chromosome 19. (B) Model chromosomes were initially arranged as in the mitotic-like configuration of Fig. 1B, but the pairings between genes were randomized. The randomization preserved the number of pairs that each probe set takes part to. (C) Model chromosomes were initially arranged as in the mitotic-like configuration of Fig. 1B, but the gene positions along the chromosome were randomized. The randomization preserved the native pairings of the genes. In all panels chromosome regions involved in the native or randomized coregulatory network are highlighted in red. For all the three systems considered the same physical conditions of fiber density, stiffness and excluded volume interactions of the original system apply.

To address these issues we re-applied the steering protocol starting from 3 different initial conditions, which correspond to specifically designed variants of the model chromosomes. Specifically, the three systems are:

1. A *random-walk-like* chromosome arrangement as shown and described in Fig. 4A.
2. A mitotic-like spatial arrangement but with *randomized gene pairings*, see Fig. 4B. The chromosome spatial configuration is the same as in Fig. 1B, but the native 1,487 coregulatory pairings between the 412 selected genes have been randomly reshuffled. The number of pairings that each selected gene takes part to in the reshuffled network is the same as the native coregulatory network.
3. A mitotic-like spatial arrangement but with *randomized gene positions*, see Fig. 4C. As in case 2 above, the chromosome spatial configuration is again the same as in Fig. 1B, but the positions of the 412 genes involved in the native coregulatory network are randomly assigned along the chromosome (except for the centromeric region). The repositioned genes inherit the native coregulatory pairings.

As for the native network of target gene pairs, we report on the properties measured at the end of the steering protocol after averaging them over the six chromosome copies in the simulation cell.

We stress that the three variants are prepared so to preserve the native overall density, number of coregulated genes and also the number of coregulated pairs to which a selected gene takes part to. They nevertheless present major differences which allow for probing the impact of different system properties on gene “colocalizability”.

**Figure 5:**
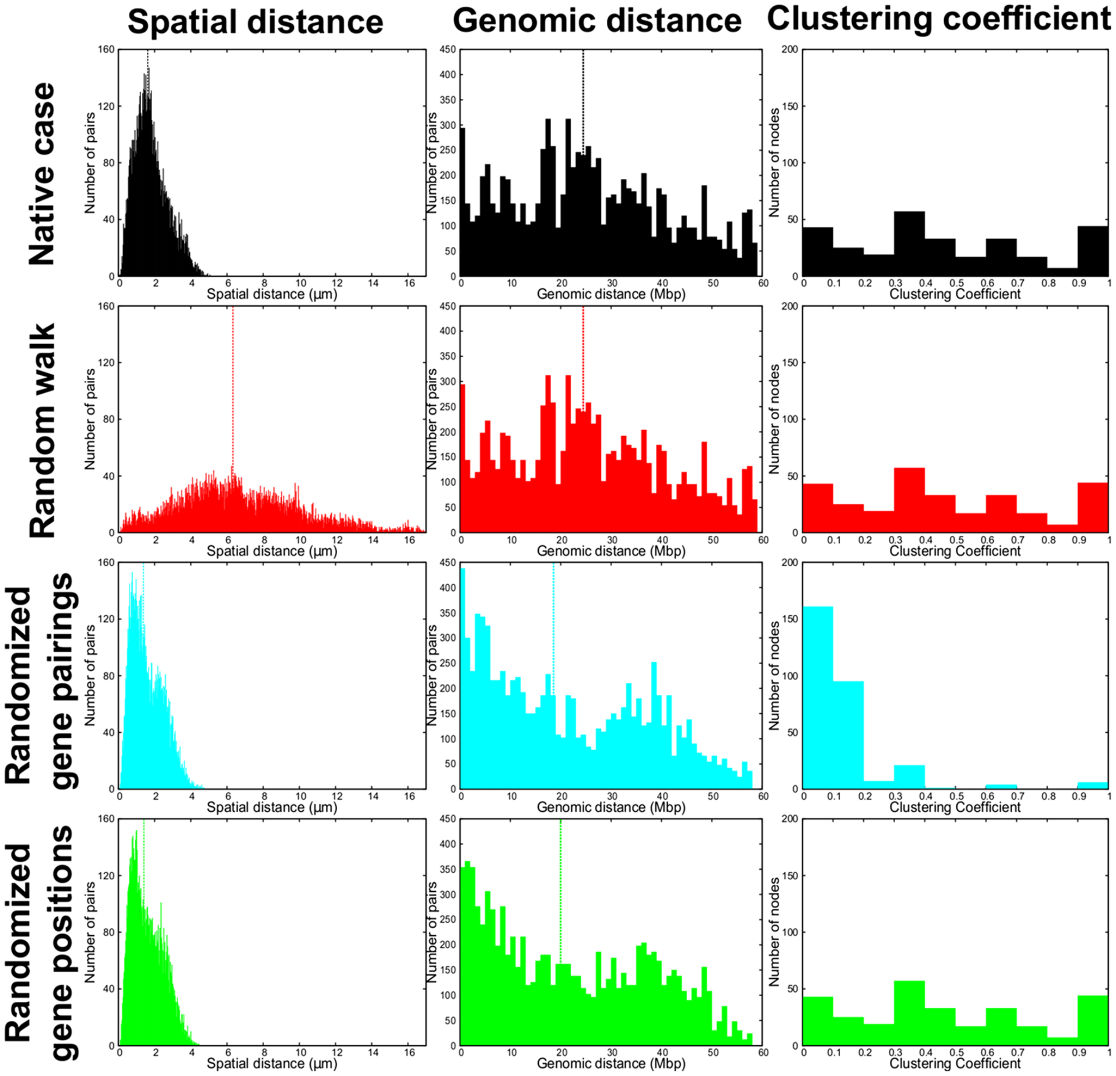
Summary of the structural properties of the native system (Fig. 1B) and its three variants (Fig. 4). (First column) Distribution of the *spatial* distances between steered *loci*. The distribution of the random-walk-like is broader than the native case one. The randomized position and randomized pairs cases have instead a similar distribution with respect to the native case. (Second column) Distribution of the *genomic* distances between steered *loci*. (Third column) Clustering coefficients (see Materials and Methods) of the corresponding networks of pairings between steered *loci*. Dashed lines correspond to the median values. The results are cumulated over all 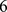 chromosome copies in the simulation box.

In particular, the random-walk-like arrangement has a much higher degree of intra- and inter-chain entanglement than all other arrangements, as illustrated by the much wider distribution of gene pairwise distances in the initial configuration, see Fig. 5. For randomly-paired and randomly-repositioned genes, instead, the distributions of genomic distances of the target genes to be paired is similar to the native one. This is clearly shown by the distributions in Fig. 5. However, the same figure clarifies that the two randomized cases differ markedly from the native one for the clustering coefficient. The clustering coefficient captures the degree of cooperativity of the (putative) coregulatory network in that it measures how frequently two genes that are both coregulated with a third one, are themselves coregulated too. The inspection of the rightmost graphs in Fig. 5 therefore indicates that the clustering coefficient distribution of the randomly-paired system is shifted towards much smaller values than the others, which all inherit the native pairings network. This fact indicates that the clustering coefficient of the native network is significantly larger than random. This implies that genes can frequently interact concertedly in groups of three or more.

**Figure 6:**
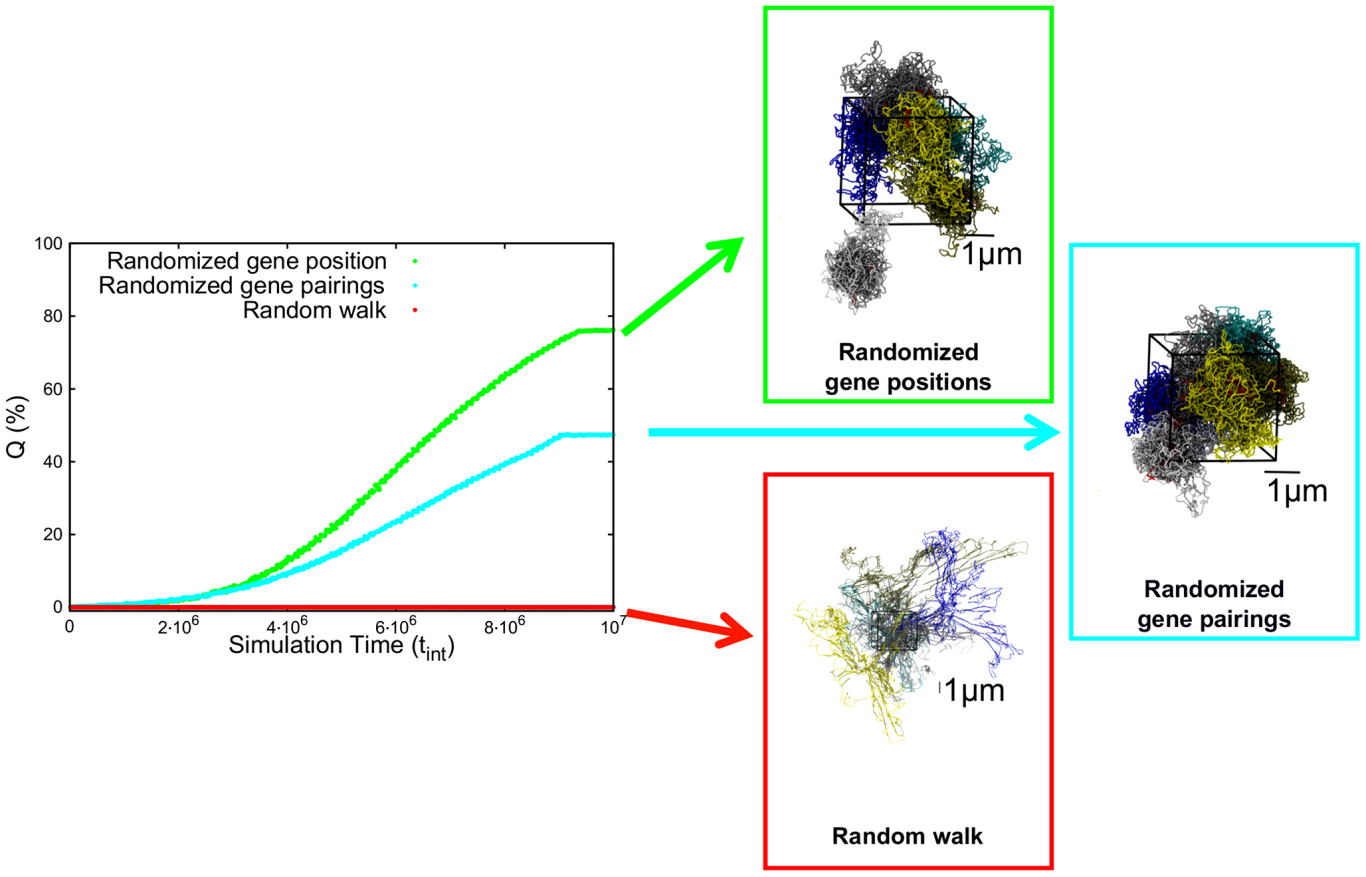
Increase of the percentage, 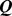, of Chr19 coregulated pairs which colocalize during the MD steering protocol, for the three variants of the native systems. The configurations reached at the end of the steering protocol are shown on the right. Chromosome regions that take part to the pairs of loci to be colocalized are highlighted in red.

The results of the steering protocol applied to the three system variants are shown in Fig. 6. The data indicate that: (i) for random-walk-like chromosomes only a minute fraction (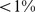) of the target contacts can be satisfied; (ii) for randomly-paired genes about 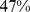 of the gene pairs can be colocalized, while (iii) for randomly-repositioned genes about 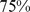 of the gene pairs can be colocalized, similarly to the native case (Fig. 2).

These findings provide valuable clues for interpreting the high degree of “colocalizability” of coregulated genes observed in Fig. 2 for the mitotic and interphase arrangements.

In particular, the very low asymptotic value of the percentage of successfully colocalized gene pairs for the random-walk-like system clarifies that the low intra- and inter-chromosome entanglement of both the mitotic and decondensed configurations is crucial for bringing into contact the coregulated gene pairs.

Furthermore, the comparison of the randomly-paired and randomly-repositioned gene cases shows that the connectivity properties of the native coregulatory network appear even more important than the detailed positioning of the coregulated genes along the chromosomes. In fact, the randomly-repositioned genes – which retain the same clustering coefficient of the native coregulatory graph – have the same high degree of colocalizability of the native system. By converse, the low clustering coefficient of the randomly-paired gene case – corresponding to a significant disruption of the original network – reflects in an appreciably lower value of percentage of successfully colocalized gene pairs. It is also worth noticing that, in all cases, a significant fraction of gene pairs brought in contact are at large genomic distances (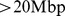), see Fig. S3.

Finally, the network randomization effects on the spatial organization of the steered conformations was addressed by measuring the overlap of their spatial macrodomains with those established from HiC data. We recall that for chromosome subdivisions into eight macrodomains, the native case overlap was 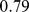. For the randomized gene positions and randomized gene pairings we instead observe the lower values 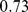 and 0.63, respectively. These values clearly have a much lower statistical significance than the native case; their 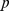-values being respectively 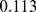 and 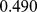, see Fig. S2. Their non-significant similarity with the reference, HiC-data based macrodomain subdivisions underscores the randomized, non-native constraints result in appreciably-different, and less realistic, chromosomal features.

### Summary and conclusions

Recent experimental advancements have provided unprecedented insight into the occurrence of concerted transcription of multiple genes. In particular, it was reported that the chromatin fiber can rearrange so that genes, concertedly transcribed upon activation, are found nearby in space too.

Because of its important ramifications, the possible existence of a general relationship between gene coregulation and gene colocalization, the so called “gene-kissing” mechanism [11],[12], is a subject of very active research.

This standing question was addressed here numerically by carrying out molecular dynamics simulations of a knowledge-based coarse-grained model of human chromosome 19. The model consisted of a coarse-grained representation (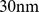 resolution) of the chromatin fiber complemented by the knowledge-based information of the loci corresponding to (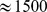) coregulated gene pairs. These pairs were identified from the analysis of extensive sets of publicly-available gene expression profiles. To mimic the crowded nuclear environment, we considered a system where several copies of the model chromosome 19 were packed at typical nuclear densities. The colocalization of the coregulated gene pairs was finally imposed by applying a steered molecular dynamics protocol.

It was found that most (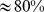) of the coregulated pairs could be colocalized in space when the steering protocol was applied to chromosomes initially prepared in mitotic-like and interphase-like arrangements, see Fig. 2. Notably, the pattern of intra-chromosome contacts established for the steered conformations exhibited significant similarities with that of experimental contact propensities [6],[7] of chromosome 19. Furthermore, the overall chromosomal organization into spatial macrodomains showed significant similarities with that inferred from experimental HiC data.

By converse, the percentage of colocalized target pairs decreased substantially (or vanished altogether) when the system was initially prepared in a random-walk like arrangement, or if the genes to be colocalized were randomly paired or displaced along the chromosome. Likewise, the macrodomain organization of these alternative systems was found to be much less similar to the HiC-data based one.

The present findings allow to draw several conclusions. First, the data in Fig. 2 demonstrate that, even in a densely packed system of mitotic or interphase chromosomes it is physically feasible to achieve the simultaneous colocalization of a large number of pairs of loci that can be very far apart along a chromosome. This result is therefore well compatible with the gene coregulation–colocalization hypothesis. In fact, the findings can be read as adding support to the hypothesis in consideration of the fact that if no meaningful relationship existed between coregulation and colocalization one might have expected the unfeasibility of bringing into simultaneous contact so many coregulated pairs.

The much poorer compliance of alternative systems (random-walk-like chromosome conformations, randomized gene pairings and positions) to the steering protocol provides valuable insight into the native chromosomal properties that allow for gene colocalization.

The first and most important property is the low degree of entanglement that mitotic or interphase chromosomes are known to have compared to equilibrated polymer solutions of equivalent density [6],[21],[24]–[28],[30],[31]. The second property is that the number of gene cliques that is present in the native gene regulatory network of chromosome 19 is much higher than for the equivalent random network. In this respect it is worth pointing out that the atypically large number of cliques found in biological regulatory networks has also been observed and pointed out in different contexts and for a different set of chromosomes [32].

To further validate this conclusion we considered an additional target network for the steered-MD simulations. This network was obtained by a partial randomization of the native gene pairings and its average clustering coefficient was 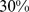, which is intermediate to the native one (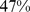) and the fully-randomized case (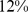) discussed previously. As shown in Fig. S4, 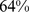 of the target colocalization constraints were satisfied. This value is intermediate between the native and fully-randomized case (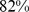 and 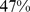, respectively) and hence supports the existence of a meaningful correlation between gene colocalizability and the regulatory network cliquishness.

In perspective, because the computational strategy employed here is formulated in a general and transferable way, it would be most interesting to apply it to other eukaryotic chromosomes for which extensive co-regulatory data is available. This could clarify the more general validity of the gene coregulation-colocalization relationship as well as the broader implications of using it (possibly with other knowledge-based constraints [20],[33],[34]), for charting the spatial organization of eukaryotic chromosomes, and possibly of systems of chromosomes.

## Materials and Methods

### Coregulated gene pairs on Chr19

To identify the set of significantly coregulated gene pairs on Chr19 we processed a set of 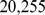 expression profiles of human probe sets measured in 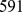 distinct microarray experiments. The gene expression profiles, which were all measured on HG-U133A Affymetrix chip, pertain to different human cell types and tissues in various experimental conditions. This extensive dataset was recently compiled and curated by some of us [10] starting from the public ArrayExpress database [35].

The analysis was restricted to the set of 1,278 probe sets which exclusively target a single sequence (i.e. an uninterrupted stretch) of chromosome 19. Next, to perform a robust comparison between the differently normalized gene expression profiles we coarse-grained all expression levels to one of three discrete states only: low, medium and high, as done in Ref. [10]. For each possible probe set pair, 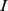 and 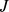, we next computed the mutual information [10] content (MI) of the expression profiles:
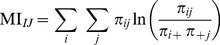
(1)where 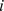 [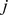] runs over the three coarse-grained expression levels for probe set 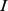 [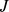]. In Eq. 1, 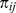 is the joint probability that, in a given experiment, the expression levels 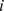 and 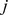 are respectively observed for probe sets 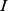 and 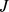, while the quantities 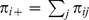 and 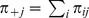 are the probabilities to observe expression level 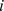 [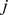] for probe set 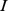 [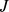] (marginal probabilities). The MI thus provides a statistically-founded measure of how the gene expression *pattern* for gene 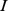 is predictable assuming the knowledge of another *pattern* 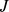 (or, vice versa).

**Figure 7:**
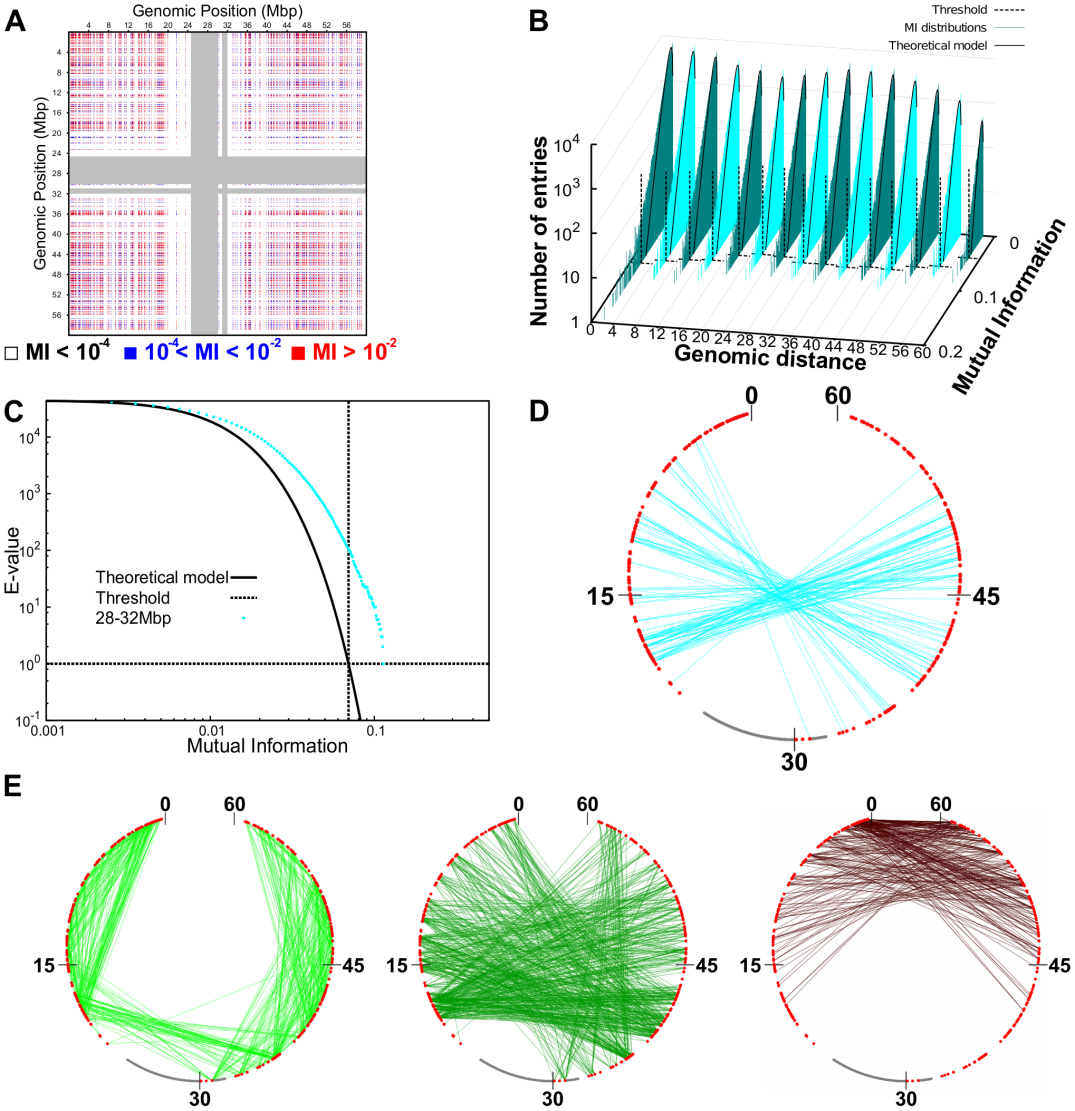
Statistical analysis of mutual information. (A) Mutual information values for any pairs of probe sets on Chr19. The middle point of each probe set identifies its position along the chromosome. The gray stripes correspond to the centromere. (B) Histograms of values of mutual information for pairs of probe sets located at various intervals of their genomic separation. The black lines correspond to fitting the histograms with the theoretical (null case) MI distribution [36]. The vertical black dashed lines correspond to the estimated threshold values (see next and main text). (C) Example of E-value (expected number of false positives) distribution for probe set pairs located at genomic separation in the range 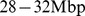. The threshold is the value of mutual information at which the E-value is equal to 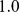. For different genomic separations, analogous curves were obtained. (D) Network of coregulated pairs of genes at 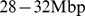 separation. The analysis illustrated in (C) singles out significantly-high values of Mutual Information. These contributions corresponds to connections (*cyan links*) between coregulated gene pairs (*red dots*). The scale is in 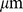. (E) Networks of coregulated pairs of loci used to fix the spatial constraints between corresponding regions of the model chromosomes. For the sake of clarity, the whole network has been represented as three sub-networks for pairs of loci at genomic separations of 0–20 Mbp (*left*), 20–40 Mbp (*middle*) and 40–60 Mbp (*right*), respectively.

To single out the pairs of probe sets with statistically-significant coexpression we proceeded according to the procedure described below and summarized graphically in Fig. 7.

First, to account for the expected dependence of gene coregulation on genomic distance, we subdivided the probe set pairs in 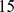 groups. The first, second, etc. group gathered pairs of probe sets whose central bases had a genomic distance falling in the intervals 0–4 Mb, 4–8 Mb, etc. Next, for each group we fitted the histogram of the pairwise MI values, with the analytical expression 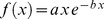 which is known to approximate well the distribution of MI values expected for two random variables (expression of the two genes) assuming 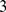 possible distinct values (low, medium and high) [36]. In the previous expression 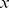 is the mutual information and 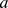 and 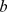 are the free fitting parameters.

The comparison with the reference, null distribution is used to define the Mutual Information threshold above which at most one false-positive entry is expected to occur. All probe set pairs exceeding this stringent MI threshold were retained (see Fig. 7C).

The number of selected pairs for each bin ranged from 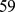 to 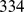, for a total of 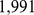 probe pairs. It should be noted that several of these pairs involve chromosome regions that are highly overlapping and are hence degenerate (or nearly degenerate). To eliminate this redundancy, we grouped together the pairs of coregulated probe sets that assure the coregulation of regions, whose central beads are separated by less than 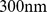 (which corresponds to the chromatin fiber statistical (Kuhn) length [21]). For each of these groups, we retained only the pair with the largest MI value. This filtering procedure returned 1,487 non-degenerate probe set pairs, that involved 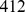 probe sets (native case). As customary, the significant degree of coexpression of such pairs was deemed indicative of their coregulation [23].

#### Randomized cases

Besides the “native case”, in which the gene pairs to colocalize are obtained from coregulatory network of Chr19, we considered another non-native set of target gene pairs. As described hereafter, these alternative sets were generated by randomizing the native gene pairing network while preserving various overall network properties.

1. *Randomized pairings*. The 1,487 native pairings between the considered set of 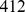 probe sets were randomly reshuffled while preserving the native number of pairings for each gene. This alternative set of probe set pairs is obtained by applying the iterative randomization method described in ref. [37]. The asymptotic fraction of randomized gene pairs matching the native ones is 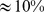.
2. *Randomized positions*. The set of 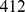 native probe sets are randomly repositioned along the contour length of the chromosome, but the target gene pairings are kept the same as the native ones. Gene repositioning in the centromeric region (which is mostly void of genes) was disallowed.

The feasibility to colocalize in space the 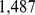 pairs of probe sets was explored using the coarse-grained model chromosome and the steering molecular dynamics protocol described in the following subsections.

### The chromosome polymer model

A system of densely packed chromosomes was modelled at a resolution of 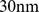. Specifically, we considered 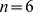 model chromosomes packed at the typical nuclear density of 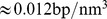. Each of the six chromatin fibers was described as a chain of 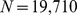 beads with diameter 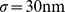, which corresponds to the total contour length 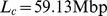 of human chromosome 19. Each bead therefore represents 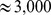 base pairs [38].

The potential energy of each chain is written as,
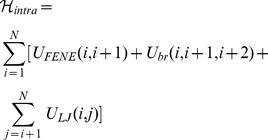
(2)where 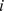 and 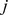 run over the bead indices and the three terms correspond to the FENE chain-connectivity interaction [39], the bending energy, and the repulsive pairwise Lennard-Jones interaction. The three energy terms are parametrized as in previous studies of coarse-grained chromosomes [21],[29]. Specifically,
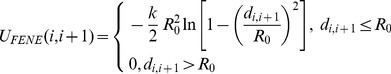
(3)where 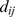 is the distance of the centers of beads 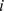 and 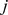, 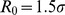, 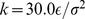 and the thermal energy 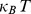 equals 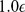 [39]. 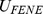 ensures the connectivity of the chain, i.e. the centers of two consecutive beads must be at a distance about equal to their diameter. The bending energy has instead the standard Kratky-Porod form (discrete worm-like chain):
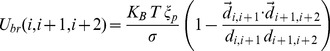
(4)where 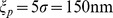. 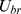 ensures that the chain of beads bends over contour lengths the size of the persistence length 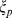 to model the experimental rigidity of the chromatin fiber [40].

Finally, the excluded volume interaction between distinct beads, including consecutive ones, corresponds to a purely repulsive Lennard-Jones potential:
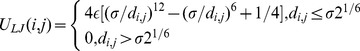
(5)This repulsive interaction controls the inter-chain excluded volume too:
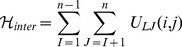
(6)where 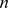 is the number of chains in solution and the index 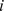 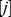 runs over the beads in chain 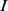 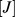. 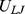 ensures that any two regions along the same chain or on different chains cannot pass through each other. In this way, intra- and inter-chain topology is preserved.

### Simulation details

The LAMMPS molecular dynamics software package [41] is used to integrate the system dynamics at constant temperature and volume. The integration time step was set equal to 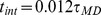, where 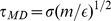 is the Lennard-Jones time and 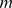 is the bead mass which was set equal to the LAMMPS default value. Periodic boundary conditions apply.

The “native case” system was evolved from three different starting conditions shown in Fig. 1: *mitotic, interphase* and *random arrangements*, whereas the *randomized cases* systems were evolved from the *mitotic* one.

#### Steered Molecular Dynamics protocol

The colocalization of the 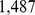 coregulated genes was attempted by using a steered molecular dynamics protocol which progressively favoured the spatial proximity of the pairs of genes in each of the six model chromosomes.

Specifically, for each pair of selected genes, 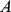 and 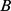, we added to the system energy an harmonic constraint,
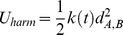
where 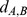 is the distance of the centers of mass of the chromosome stretches (mapped onto the discrete beads using the Affymetrix annotation table (http://www.affymetrix.com)) covered by the two genes. The stiffness of the harmonic constraint was controlled by the time-dependent parameter 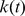. The latter is ramped linearly in time from the initial value 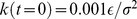 up to the value 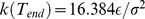. The total duration of the steered dynamics was 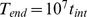. This protocol favours the progressive reduction of the width of the distribution of probe set distances from the initially generous value of 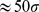 (see 5) down to 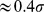. The simultaneous application of the 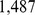 constraints to each of the six chromosomes, which clearly are not necessarily compatible *a priori*, was implemented using the PLUMED plugin for LAMMPS [42]. The protocol is sufficiently mild that no crossings of the chains should occur. This was checked by running the steering protocol on a circularized variants of the mitotic conformation shown in Fig. 1A, and checking that the initially unknotted topological state is maintained [43].

### Order parameters

To monitor the progress of the steered molecular dynamics simulations and to characterize the salient properties of the resulting configurations we computed two order parameters, namely the *percentage of coregulated pairs that are colocalized* and the *clustering coefficient* of the coregulated pair graph. The two parameters are defined hereafter.

- The *percentage of coregulated pairs that are colocalized*, 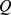, is calculated as:
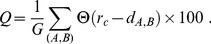
(7)

In the above expression, the sum runs over the coregulated pairs of genes, 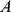 and 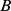 which are in total 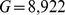 (i.e. 1,487 for each of the six chromosome copies), 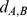 is the distance of their centers of mass. 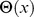 is the Heaviside step which takes a value of 1 if 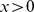 and 0 otherwise. 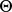 is used to restrict the sum to those gene pairs that are at distance within the contact range, 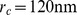. This cutoff distance was chosen because it is about equal to the typical size of a “transcription factory” [19].

- The *clustering coefficient*, 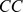, is used to characterize connectivity properties of graphs. In the present case the graph of coregulation of pairs of genes. Each gene is represented by a node in the graph. Pairs of coregulated genes are represented by a link connecting the two corresponding nodes.

The clustering coefficient of the individual 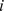
th node in the graph is defined as [44],[45]

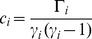
(8)where 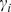 is the number of neighbours of 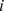 while 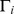 is the number of distinct links between the neighbours of node 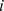. The clustering coefficient per node, 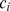, is clearly defined only for nodes with at least two neighbours. The clustering coefficient of the whole graph is obtained by averaging 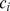 over all nodes with 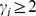. The clustering coefficient provides a measure of the incidence of cliques of size 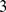 (“triangular linkages”) in the graph.

### Identification of spatial macrodomains

The overall spatial organization of Chr19 was encoded in a binary contact matrix, 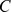, with a 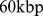 resolution. The generic matrix entry 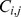 takes on the value 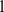 or 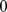 according to whether the 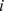
th and 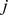
th 60kbp-long segments (equivalent to 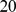 beads) are in spatial proximity or not. The recent high-resolution HiC measurements of Dixon et al. [9] were used to derive the experimental, reference contact map. Specifically, for every significant HiC entry (i.e. normalized contact enrichment 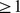) the corresponding contact-matrix elements were set equal to 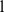. The resulting HiC-based contact map is sparse in that only 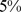 of its entries are non-zero. For an equal footing comparison, we next populated the theoretical contact maps by considering in spatial contacts (entries equal to 1) only the top 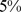 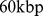-strands ranked for increasing average distance. The distance average is taken over the six Chr19 copies at the end of the steering protocol.

A clustering analysis of the contact maps was next used to subdivide Chr19 into up to ten spatial macrodomains. Each domain spans an uninterrupted stretch of the chromosome and one domain always matches the centromere region. Following the K-medoids clustering strategy [46] the optimal domain partitioning was identified by minimizing the total intra-domain dissimilarity. Quantitatively, the internal dissimilarity of one specific domain, covering the chain interval 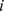 to 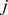 is measured as:
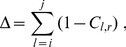
(9)where 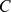 is the contact map and 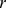, which is the domain representative, is the element belonging to the 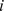
–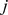 interval for which 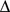 is minimum. Consistently with intuition, the dissimilarity score, 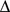, takes on small or large values if respectively many or few domain members are in contact with the representative. For a given number of domains, the optimal domain partitioning is the one that minimizes the sum of the 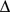 scores for the domains.

For a given number of domains, the consistency of the steered-MD and HiC-based subdivisions was measured by establishing a one-to-one correspondence of each domain in the two cases and next measuring the percentage of elements, 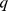, having identical domain assignment. The one-to-one domain correspondence was identified by exploring the combinatorial space of correspondences and picking the one yielding the largest value of 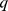.

## Supporting Information

Figure S1
**Chr19 spatial macrodomains.** The filled circles mark the boundaries of the Chr19 spatial macrodomains obtained from the clustering analysis of the steered-MD contact maps (top) and inferred from HiC data (bottom). The number of imposed macrodomains is shown on the 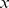 axis. In all cases, one domain was fixed to correspond to the centromere (for which no HiC data are available) which is shown in grey. The dashed guidelines mark the subdivision into eight macrodomains which, by visual inspection provides robust, consensual boundaries in both cases. For clarity, the eight-domain subdivision is also reported on the chromosome sketch on the right.(TIF)

Figure S2
**Comparison of macrodomain subdivisions.** (A). Schematic representation of the Chr19 partitioning in 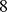 macrodomains (one being the centromere) based on the clustering analysis of contact maps inferred from HiC data and from steered-MD simulations on the native and randomized versions of the gene pairing network. In all cases, one domain was constrained to match the centromere (shown in grey). The overlap, 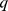 and associated 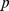-value of the steered-MD subdivisions against the reference HiC-data based one are as follows, (i) native case: 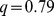, 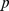-value = 0.027; (ii) randomized gene positions: 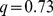, 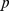-value = 0.113; (iii) randomized gene pairings: 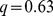, 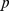-value = 0.49. The 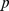-values were computed by comparing the observed overlap against a reference distribution of overlaps of 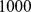 random chromosome partitions into 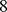 domains (one always corresponding to the centromere). The reference distribution is shown in panel B. The arrows indicate the overlaps of the native and randomized cases.(TIF)

Figure S3
**Genomic distance distribution for the target gene pairings established at the end of the steering protocol.** The plots on the left provide the genomic distance distributions of target gene pairings that are actually satisfied at the end of the steering protocols for the native and randomized cases. The analogous distribution for non-satisfied pairings is shown on the right. Dashed lines correspond to the median values. The results are cumulated over all 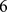 chromosomes copies in the simulation box.(TIF)

Figure S4
**Gene colocalizability and gene network cliquishness. The time evolution of the fraction of satisfied gene pairings for three different steered-MD simulations.** The target gene pairing networks for the simulations are: the native network and two variants of it obtained by partial and full randomizations of gene pairings. The curves for the native and fully-randomized cases are the same as in Fig. 6. The different cliquishness of the three target networks is captured by their clustering coefficient: 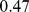 for the native case, 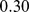 for the partially-randomized case and 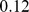 for the fully-randomized case. The fraction of established pairings shows a clear monotonic (increasing) dependence with the clustering coefficient.(TIF)
