# Supplementary figures and images for: Colocalization of Coregulated Genes: A Steered Molecular Dynamics Study of Human Chromosome 19
Source: PLoS Comput Biol. 2013 Mar 28;9(3):e1003019. doi: 10.1371/journal.pcbi.1003019 (PMC3610629; doi:10.1371/journal.pcbi.1003019)

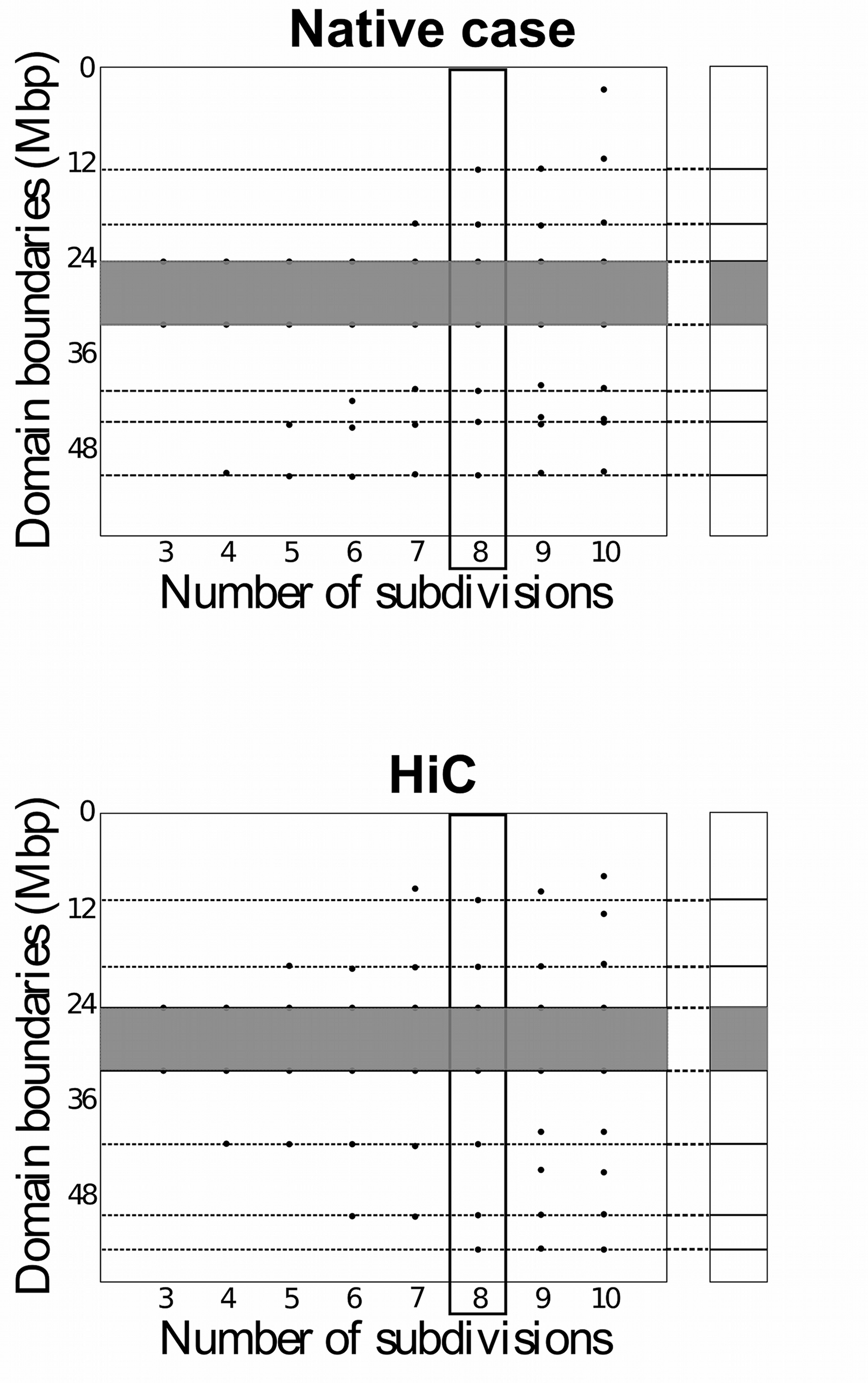

Supplement: Figure S1 — Chr19 spatial macrodomains. The filled circles mark the boundaries of the Chr19 spatial macrodomains obtained from the clustering analysis of the steered-MD contact maps (top) and inferred from HiC data (bottom). The number of imposed macrodomains is shown on the axis. In all cases, one domain was fixed to correspond to the centromere (for which no HiC data are available) which is shown in grey. The dashed guidelines mark the subdivision into eight macrodomains which, by visual inspection provides robust, consensual boundaries in both cases. For clarity, the eight-domain subdivision is also reported on the chromosome sketch on the right. (TIF) [file pcbi.1003019.s001.tif]

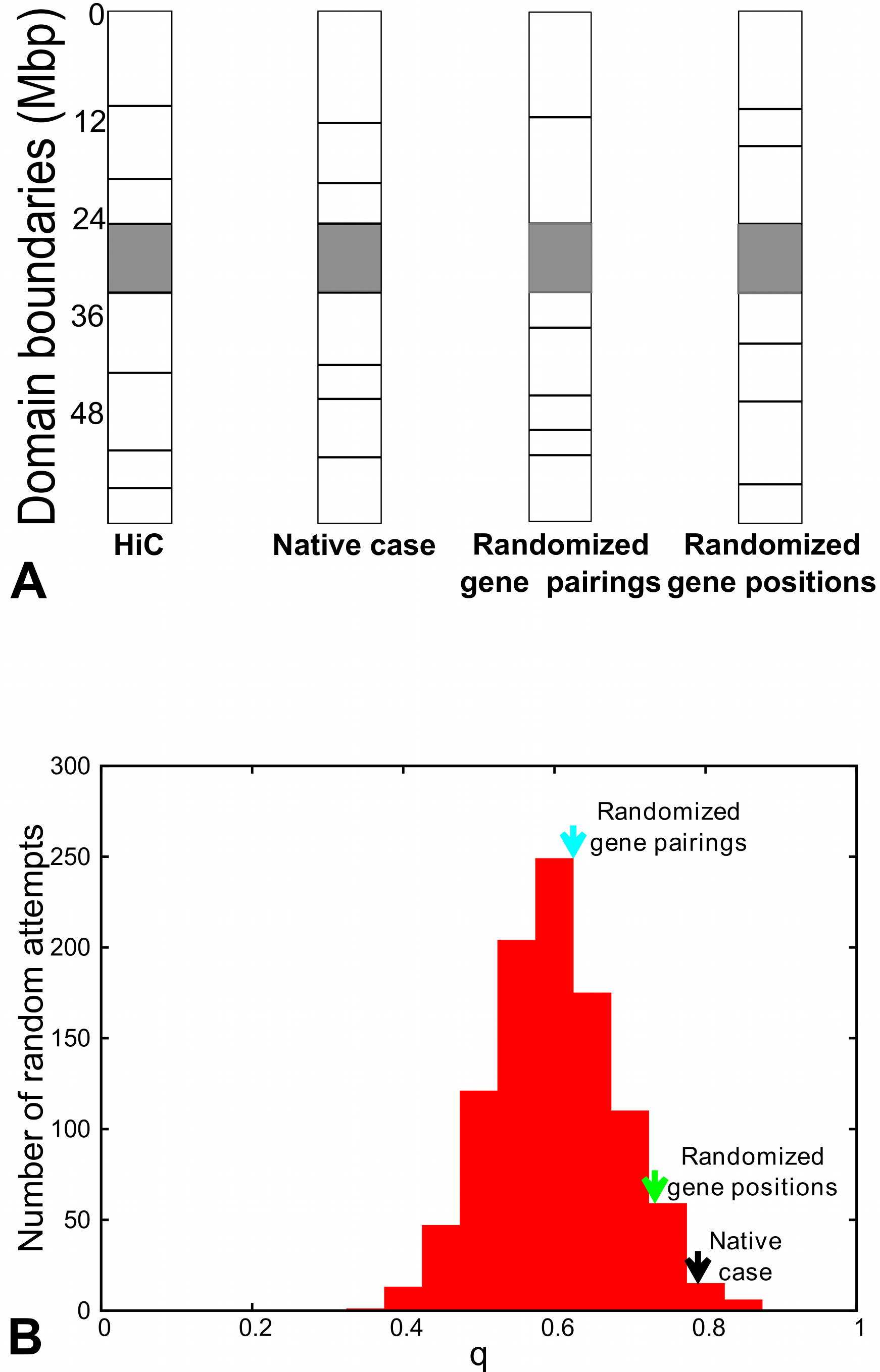

Supplement: Figure S2 — Comparison of macrodomain subdivisions. (A). Schematic representation of the Chr19 partitioning in macrodomains (one being the centromere) based on the clustering analysis of contact maps inferred from HiC data and from steered-MD simulations on the native and randomized versions of the gene pairing network. In all cases, one domain was constrained to match the centromere (shown in grey). The overlap, and associated -value of the steered-MD subdivisions against the reference HiC-data based one are as follows, (i) native case: , -value = 0.027; (ii) randomized gene positions: , -value = 0.113; (iii) randomized gene pairings: , -value = 0.49. The -values were computed by comparing the observed overlap against a reference distribution of overlaps of random chromosome partitions into domains (one always corresponding to the centromere). The reference distribution is shown in panel B. The arrows indicate the overlaps of the native and randomized cases. (TIF) [file pcbi.1003019.s002.tif]

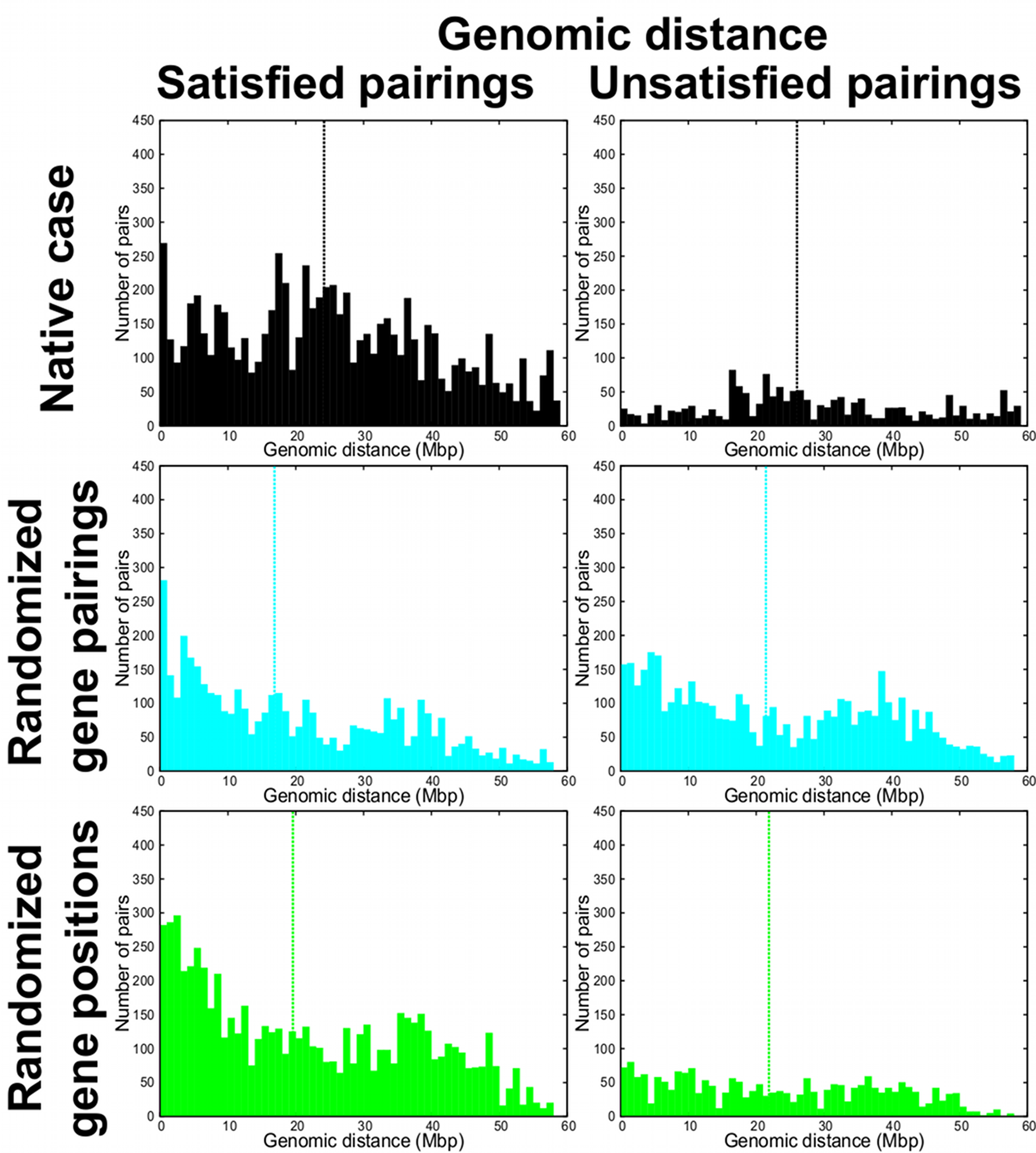

Supplement: Figure S3 — Genomic distance distribution for the target gene pairings established at the end of the steering protocol. The plots on the left provide the genomic distance distributions of target gene pairings that are actually satisfied at the end of the steering protocols for the native and randomized cases. The analogous distribution for non-satisfied pairings is shown on the right. Dashed lines correspond to the median values. The results are cumulated over all chromosomes copies in the simulation box. (TIF) [file pcbi.1003019.s003.tif]

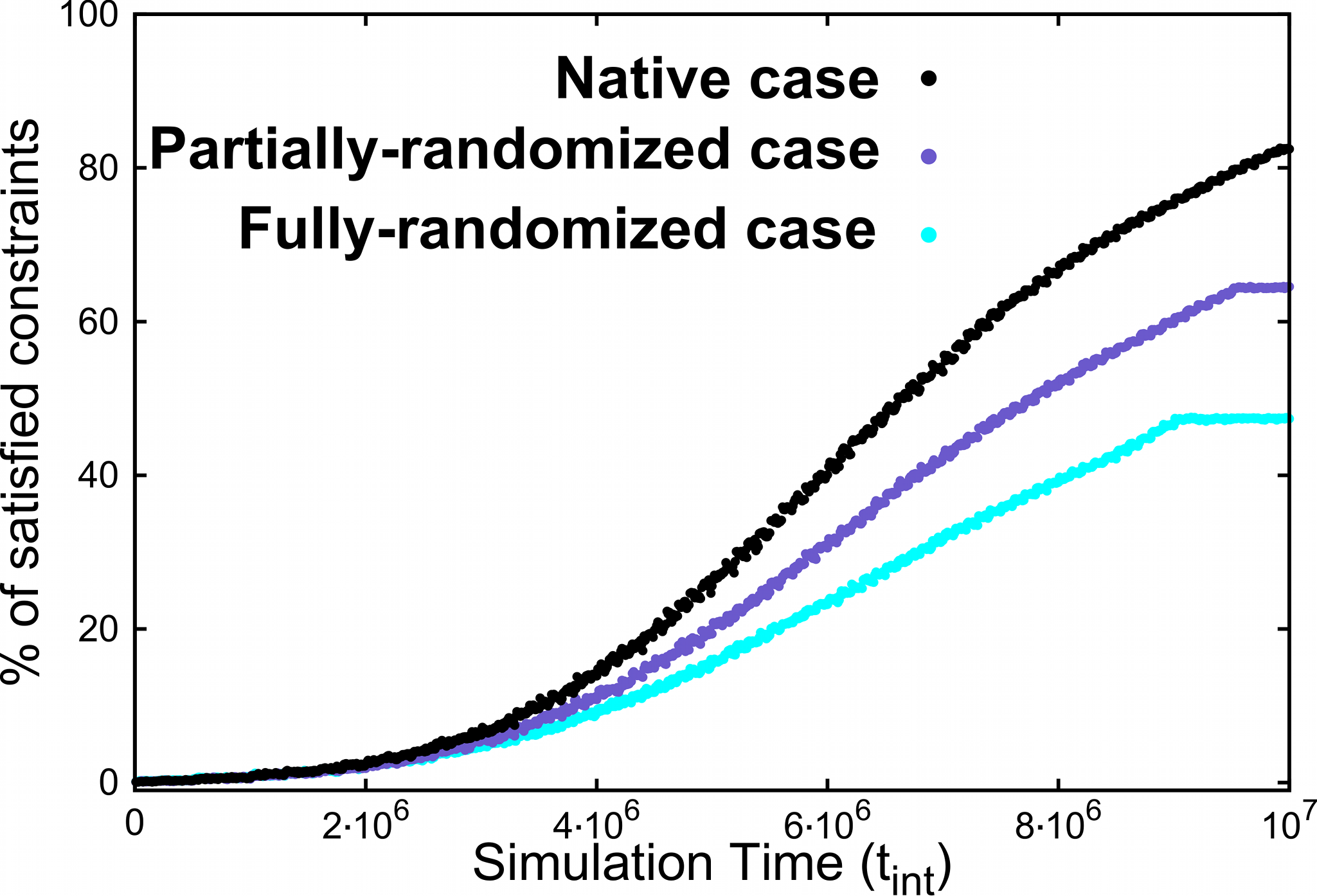

Supplement: Figure S4 — Gene colocalizability and gene network cliquishness. The time evolution of the fraction of satisfied gene pairings for three different steered-MD simulations. The target gene pairing networks for the simulations are: the native network and two variants of it obtained by partial and full randomizations of gene pairings. The curves for the native and fully-randomized cases are the same as in Fig. 6. The different cliquishness of the three target networks is captured by their clustering coefficient: for the native case, for the partially-randomized case and for the fully-randomized case. The fraction of established pairings shows a clear monotonic (increasing) dependence with the clustering coefficient. (TIF) [file pcbi.1003019.s004.tif]
